# Undiagnosed prediabetes in Mexican adolescents under poverty in contexts affected by collective violence: A clinical comparison among health services users and hidden population

**DOI:** 10.3389/fnut.2022.1007781

**Published:** 2022-11-21

**Authors:** Dewi Hernández-Montoya, Elsie E. Cedillo-Escobar, Marcelino Esparza-Aguilar, Abril Violeta Muñoz-Torres

**Affiliations:** ^1^Unidad de Investigación en Epidemiología, Instituto Nacional de Pediatría, Ciudad de México, México; ^2^Departamento de Salud Pública, Facultad de Medicina, Universidad Nacional Autónoma de México, Ciudad de México, México

**Keywords:** poverty, hidden population, adolescents, healthcare services access, prediabetes, collective violence, privacy, social determinants of health (MeSH)

## Abstract

**Introduction:**

The epidemiological pattern of prediabetes in adolescents is understudied. In Mexico, adolescents are exposed to social adversity conditions, including poverty and violence. Therefore, understanding their clinical profiles and how the social determinants of health impose barriers to access to health services is important to address detection, in those who, by their vulnerability, remain a hidden population.

**Aim:**

This study aimed to describe undiagnosed prediabetes in Mexican adolescents under poverty in violent contexts and to compare the clinical features among health services users and hidden population.

**Methods:**

This cross-sectional study included 371 adolescents from difficult access locations in violent contexts. Poverty, lack of health services access, and perceived vulnerability were determined in all samples. Endocrine markers (BMI, HOMA-IR, HbA1c, and cortisol) were measured in those with high violence perception.

**Results:**

A total of 61.7% of the adolescents had a suburban grid and urban cluster residence, and 77.7–85.7% of them belonged to locations where 35–50% of their population lived below the poverty line. In total, 40–75% had a lack of 10–20% access to health services, and 18.8% had a high perceived vulnerability due to collective violence and were screened. Overall, 61.9% of respondents were newly diagnosed with prediabetes and showed the worst HbA1c (*p* = 0.001) compared to the health services subsample, which showed the highest BMI (*p* = 0.031) and insulin resistance (*p* = 0.025).

**Conclusion:**

There is a prediabetes hidden population living in violent contexts under poverty. These social determinants promote poor outcomes in perceived vulnerability and endocrine response and represent barriers to access to health services.

## Introduction

Type 2 diabetes mellitus (T2DM) has been showing an increasing incidence in adolescents (1, 2). Impaired glucose tolerance (prediabetes hereinafter) was more prevalent than expected in the global, Latino, and Mexican populations, in which between 3.3 and 14% were undiagnosed (3). Although there are some approaches to estimate the frequency of prediabetes of 8.5 to 14.3% in Mexico, there is a gap in the study of the occurrence, distribution, and social features of the population that would define its epidemiological pattern (4–7).

Adolescents are the most exposed to conditions of social adversity (8). The association of the occurrence of diabetes, as well as its attributable burden, with increasing poverty, low income, and education among youth is well-known. In addition, early onset is favored by intermediate determinants such as income, housing, and food security (9, 10). These social exclusion conditions lead the population to a situation of helplessness and alter the way they experience healthcare (11). In Mexico, this population is also vulnerable to social problems, such as poverty, crime, and violence (12–14). Collective violence is a structural determinant affecting access to and reach of health services. Prevention, early detection, and treatment of different health-related conditions must address the social determinants that impact this access needs to be improved (15).

The Commission on Social Determinants of Health places the health system as an intermediate determinant that shows distinguished functioning between different contexts (16, 17). Suburban and rural contexts display poor conditions of marginality and deprivation, compared to urban contexts, which favor the emergence of practices such as violence and criminality, which in turn, influence the behavior and social perceptions about healthcare in these contexts (18, 19).

A hidden population is a group that is difficult to access, with no sampling frame, and the public acknowledgment of their population membership is potentially threatening. This vulnerable population underutilizes health services and remains a hidden population, since they may be linked in some way to collective violence. The hidden population was imposed barriers to accessing health services because society stigmatizes, devalues, and marginalizes them (20, 21).

It is important to describe adolescents with prediabetes who cannot be reached by the health system and who in the short term will contribute to the burden of diabetes. It is necessary to improve the diagnosis, timely treatment, and follow-up to avoid the progress of this condition and its complications. This study aimed to describe undiagnosed prediabetes in Mexican adolescents under poverty in violent contexts and to compare the clinical features among health service users and the hidden population.

## Materials and methods

### Design

This is a cross-sectional and analytical study.

### Setting

The research was conducted in 2018 at public schools from an urban center, suburban grid cells, and an urban cluster in contexts affected by collective violence (where homicide rates were within the first national quintile in states in the north and center of Mexico in 2017) (22). The recruited adolescents studied the lower secondary level according to UNESCO classification (23) and lived in all demographic areas, including rural communities (24). The fieldwork was coordinated with the Mexican Ministry of Health in the primary care services and schools through the “Salud en tu Escuela” program (25). These contexts were selected because they had higher incidences of T2DM in adolescents from 2003 to 2017. Moreover, there are entities in which 30 to 50% of the population lived in poverty during the period of the study (26). In addition, the collective violence in these states has historically been a structural determinant (27–30). Regarding health services, the urban center context had public healthcare services for the entire population with and without insurance, a social security system for the workforce population, as well as private services. In contrast, suburban grid and urban cluster contexts showed a low density of health facilities and only had the availability of one healthcare center “T1 units,” which offers a scarcity of pharmacy and basic assistance of outpatient medical and nursing care, but auxiliary diagnostic studies are not available. Furthermore, there is no health unit aimed at the worker sector, and the number of appropriately qualified health professionals is insufficient.

### Study size

**Figure 1:**
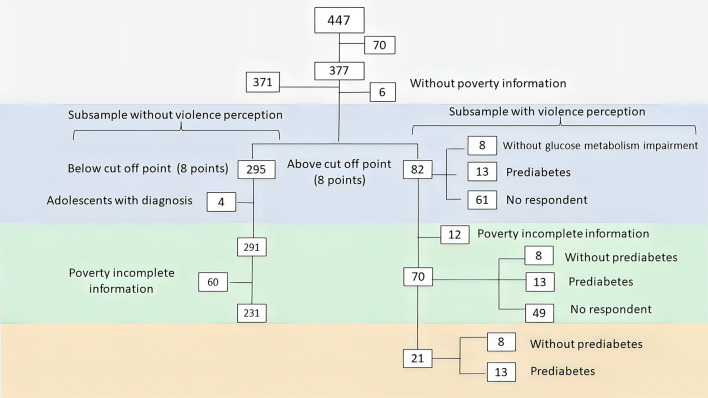
Sample selection flowchart.

This was a non-probability convenience sampling, as we identified a hidden population which, by definition, lacks a sampling frame of reference, and it is not possible to estimate the sample size because neither the actual population size nor the size of the effect of interest to be detected is known. The sample was integrated of Mexican adolescents aged 13–17, eighth graders. In the first stage, all adolescents were included and screened according to their perceived vulnerability due to collective violence. In the second stage, all adolescents above the cut-off point (8 points) were contacted for a clinical interview, and a blood sample was taken for endocrine markers. The comparative sample was contacted and enrolled from the records of the epidemiological surveillance system (Figure 1). We approached some contexts where there is only one school, in some of the suburban grid and urban clusters, and we included all eighth-grade students. The urban school was selected because of its more privileged social conditions than those available.

### Variables

The first stage included the proportions of urban poverty and lack of access to health services, as well as perceived vulnerability due to social adversity for the entire sample. In the second stage, health service coverage, exposure to violence, and endocrine markers were measured in the subsample with perceived vulnerability due to collective violence (Supplementary 1).

#### First stage

Data on residence poverty and health services in adolescents' neighborhoods were obtained from the records of the National Council for the Evaluation of Social Development Policy (CONEVAL).

***Urban poverty (poverty) *** was measured and calculated by CONEVAL according to the proportion of the population living below the national poverty line, corresponding to indicator 1.2 of the UN sustainable development goals (31, 32). CONEVAL classifies the poverty into 4 ranks (0–18, 18–34, 35–50, and 51–70%). A rank of poverty was assigned to each participant according to her/his residential neighborhood. The classification of locations followed the UN-Habitat method for the degree of urbanization as an urban center, suburban grid cell, and urban cluster (24).

***Healthcare access *** was measured as the proportion of the population lacking access, by following the same methodology and in parallel with poverty (32). A rank of healthcare access was assigned for each participant according to her/his neighborhood.

***Perceived Vulnerability (vulnerability) *** was operationalized according to the participant's report on the extent (5-point Likert scale) of social and contextual deprivation and collective violence *(**violence*** hereinafter*)* affecting their community, family, and themselves. Social deprivation included income, food security, health services, and education for their family and themselves. Contextual deprivation included health, education, and security services in the community. Finally, the violence section included the occurrence of violence and security protection by the state. Possible scores ranged from 0 to 40 and 0 to 20 for social and contextual deprivation, respectively, and 0 to 20 for collective violence. Adolescents with a vulnerability due to violence above the first SD of the sample mean (8 points) were selected for the second stage.

#### Second stage

***Socio-demographic information*** included information on their health services and social program coverage.

***Endocrine markers*** were measured in a blood sample after an overnight fast of 8–12 h. HbA1c, fasting glucose, insulin, and serum cortisol were determined. Anthropometric measurements were obtained from the school register at the time of our visit. The Homeostatic Model Assessment for Insulin Resistance (HOMA-IR) and the Body Mass Index (BMI) were calculated.

***Violence exposure*** was operationalized as either with or without exposure, according to participant's report of the regular experience of collective violence in their neighborhood.

### Integration of analytical sub-samples

In terms of clinical features, the analysis was performed by stratifying the sample into four subsample groups:

1. “***No perception of violence***”: those with a score of < 8 points among the whole sample (*n* = 231).
2. “***With a perception of violence surveyed***“: those with a score of > 8 points among the subsample, who respond to the call and were found to be without prediabetes (*n* = 8).
3. “***With the perception of violence with prediabetes***”: those with a score of > 8 points among the subsample, who respond to the call and were found with prediabetes (*n* = 13).
4. “***With the perception of violence not surveyed***”: those with a score of > 8 points among the subsample, who did not respond to the call, and glucose impairment is unknown (*n* = 61).

Regarding comparative analysis, it displayed three principal groups of adolescents with prediabetes who were stratified by violence exposure as below:

1. “***Health services users with prediabetes***” [with (*n* = 1) and without (*n* = 3) violence exposure].
2. “***All newly diagnosed participants in school***” [with (*n* = 12) and without (*n* = 6) violence exposure]. In the supplementary material, we stratified this group by origin, considering those who enrolled in screening from the schools discussed in the first stage, and those who enrolled from other schools that did not follow the described screening. We included these participants to expand the sample size and to favor the characterization of clinical profiles. We considered this sick population as a hidden population in the healthcare system.
3. “***School participants without prediabetes***” [with (*n* = 16) and without (*n* = 3) violence exposure].

Health services users with T2DM were included to show their features but were left out of the comparative statistical analysis.

### Bias

Selection bias would have to be considered since the study included adolescents or groups of adolescents who, although very similar to each other, did not share the characteristics of the general population from which they were selected. This sample represents a group of adolescents living in hard-to-reach places, where poverty and violence act as structural problems. Likewise, there were some missing values of endocrine markers, which were not missing at random. The probability of a missing value was dependent on the observed barriers related to the healthcare accessibility of the study subjects. In addition, the results of the study were derived from data collected *a priori*, and the evidence was the result of an empirical finding in the field.

### Statistical analysis

#### First stage

A descriptive analysis was conducted to determine the proportion of the whole sample living in poverty according to the CONEVAL classification and to the degree of urbanization, as well as the proportion lacking access to health services. Statistical differences between groups (poverty ranks) were determined using the Chi-square test. Medians and quartiles of BMI and vulnerability scores were estimated, and statistical differences between groups were determined using the Jonckheere-Terpstra test because poverty has an *a priori* ordering of the population from which the subsamples were drawn. For the comparison between subsamples according to their vulnerability and response to the call, Mood's median test was used.

#### Second stage

The response rate and the proportion of adolescents with prediabetes and the proportion that remains hidden from the health system were calculated. Moreover, we estimated health service coverage proportions, medians, and quartiles for endocrine markers. We also tested for differences between health services users and hidden population groups by level of measurement using a Chi-square and the Kruskal-Wallis tests, respectively.

All the tests were performed with IBM SPSS Statistics for Windows, Version 28.0. We established a confidence level of 95% for all statistical tests.

## Results

The sample consisted of 371 adolescents aged 13–17 years (median = 13) and 197 (53.2%) were female. The differences between groups were only statistically significant in age (*p* < 0.001), and those who kept their data privacy showed the highest median at 14 years. Among the whole sample, 300 (80.8%) lived in some urban area; 101 (27.3%) were distributed by urban neighborhoods, mainly those with a 35–50% of poverty proportion; 32 (9.5%) lived in rural areas, and 34 (9.2%) preferred to keep their data private. The households with the lower poverty were in the urban center and, in contrast, those with the higher poverty were in the suburban grid and the urban cluster (*p* < 0.001). Most adolescents lived in urban contexts where the lack of health services among their inhabitants reached between 10 and 20%, regardless of poverty, although those living in rural areas showed a higher proportion (Table 1).

**Table 1:** Sociodemographic features by urban poverty sector among the sample.

		**Urban poverty proportion**				**Rural**	**Keep housing data private**	
	**Sample distribution**	**0–18%**	**19–34%**	**35–50%**	**51–70%**			***p*-Value Chi-square test**
		**Lower**			**Higher**			
	***n* (%) of total and relative sample or median** ** (Q1, Q3)**	***n* (% of catregory) or median** ** (Q1, Q3)**	***n* (% of catregory) or median** ** (Q1, Q3)**	***n* (% of catregory) or median** ** (Q1, Q3)**	***n* (% of catregory) or median** ** (Q1, Q3)**	***n* (% of catregory) or median** ** (Q1, Q3)**	***n* (% of catregory) or median** ** (Q1, Q3)**	
**Total and relative samples**								
n All sample	*n* = 371 (100%)	78 (21.1%)	69 (18.6%)	101 (27.3%)	52 (14.1%)	35 (9.5%)	34 (9.2%)	
n1 Subsample with collective violence perception	*n* = 70 (18.8) % n	16 (22.9%)	13 (18.6%)	23 (32.9%)	7 (10.0%)	2 (2.9%)	8 (11.4%)	
n2a Respondent	*n* = 21 (30.0) % n1	2 (9.5%)	3 (14.3%)	9 (**42.9%**)	4 (19.0%)	2 (9.5%)	1 (4.8%)	
n3 Respondent with prediabetes	*n* = 13 (61.9) % n2a	2 (15.4%)	2 (23.1%)	5 (38.5%)	2 (15.4%)	1 (7.7%)		
n2b No respondent	*n* = 49 (70.0) % n1	14 (28.6%)	10 (20.4%)	14 (28.6%)	3 (6.1%)		7 (14.3%)	
**A. Sociodemographics**		*n* (%)	*n* (%)	*n* (%)	*n* (%)	*n* (%)	*n* (%)	
**1. Sex (female)**								
All Sample	*n* = 197 (53.2) % n	34 (43.6%)	37 (53.6%)	61 (59.8%)	29 (55.8%)	14 (41.2%)	22 (64.7%)	0.116
Subsample with collective violence perception	*n* = 39 (55.7) % n1	7 (43.8%)	7 (53.8%)	16 (69.6%)	2 (28.6%)	1 (50.0%)	6 (75.0%)	0.293
1.1 Respondent	*n* = 11 (52.4) % n2a	2 (100%)	2 (66.7%)	5 (55.6%)	1 (25.0%)	1 (50.0%)		0.492
A1.1.1 Respondent with prediabetes	*n* = 9 (69.2) % n3	2 (100%)	2 (66.7%)	4 (80.0%)		1 (100%)		0.191
A2.2 No respondent	*n* = 28 (57.1) % n2b	5 (35.7%)	5 (50.0%)	11 (78.6%)	1 (33.3%)		6 (85.7%)	0.081
**2. Age**								
All Sample	Median = 13.0 (Q1 = 13, Q3 = 14)	13.0 (13.0, 13.8)	13.0 (13.0, 14.0)	13.0 (13.0, 14.0)	13.0 (13.0, 14.0)	13.0 (13.0, 13.8)	14.0 (13.0,14.0)	< 0.001
2.1 Subsample with collective violence perception	Median = 13.0 (Q1 = 13.0, Q3 = 14.0)	13.0 (13.0, 14.0)	14.0 (13.0, 14.0)	13.0 (13.0, 14.0)	13.0 (13.0, 14.5)	13.0 (13.0,13.0)	14.0 (13.3, 14.0)	
2.1.1 Subsample with prediabetes	Median = 13.0 (Q1 = 13.0, Q3 = 13.0)		13.0 (13.0, 13.5)	13.0 (13.0, 13.0)	13.0 (13.0, 13.0)			
2.2 No respondent	Median = 13.0 (Q1 = 13.0, Q3 = 14.0)	13.0 (13.0, 14.0)	14.0 (13.0, 14.0)	13.5 (13.0, 14.0)	13.0 (13.0, 14.5)		14.0 (14.0, 14.0)	
**3. Urbanistic household classification**								< 0.001^*^
3.1 Urban								
All sample	*n* = 111 (29.9%)	68 (87.2%)	40 (58%)	3 (2.9%)				
3.1.1 Subsample with collective violence perception	*n* = 20 (28.6%)	12 (75.0%)	8 (61.5%)					
3.1.1.1 Subsample with prediabetes	*n* = 4 (30.8%)	2 (100 %)	2 (66.7%)					
3.1.2 No respondent	*n* =16 (32.7%)	10 (71.4%)	6 (60.0%)					
3.2 Suburban								
All sample	*n* = 130 (35.1%)	10 (12.8%)	28 (40.6%)	80 (77.7%)	12 (23.1%)			
3.2.1 Subsample with collective violence perception	*n* = 27 (38.6%)	4 (25.0%)	4 (30.8%)	16 (69.6%)	2 (28.6%)			
3.2.1.1 Subsample with prediabetes	*n* = 3 (23.1%)			3 (60.0%)				
3.2.2 No respondent	*n* = 22 (46.9%)	4 (28.6%)	4 (40.0%)	12 (85.7%)	2 (66.7%)			
3.3 Urban center								
All sample	*n* = 62 (16.7%)		1 (1.4%)	21 (19.4%)	40 (76.9%)			
3.3.1 Subsample with collective violence perception	*n* = 13 (18.6%)		1 (7.7%)	7 (30.4%)	5 (71.4%)			
3.3.1.1 Subsample with prediabetes	*n* = 5 (38.5%)		1 (33.3%)	2 (40.0%)	2 (100%)			
3.3.2 No respondent	*n* = 3 (6.1%)			2 (14.3%)	1 (33.3%)			
**4. Lack of health services access by residence**								< 0.001^*^
4.1 0–10 %								
All sample	*n* = 68 (18.3%)	9 (11.5%)	21 (30.4%)	37 (35.9%)	1 (1.9%)			
4.1.1 Subsample with collective violence perception	*n* = 16 (22.9%)	3 (18.8%)	4 (30.8%)	9 (39.1%)				
4.1.1.1 Subsample with prediabetes	*n* = 3 (23.1%)	1 (50.0%)	1 (33.3%)	1 (20.0%)				
4.1.2 No respondent	*n* = 12 (24.5%)	2 (14.3%)	3 (30.0%)	7 (50.0%)				
4.2 11–20%								
All sample	*n* = 180 (45.8%)	37 (47.4%)	30 (43.5%)	62 (60.2%)	51 (98.1%)			
4.2.1 Subsample with collective violence perception	*n* = 36 (51.4%)	9 (56.3%)	6 (46.2%)	14 (60.9%)	7 (100%)			
4.2.1.1 Subsample with prediabetes	*n* = 9 (69.2%)	1 (50.0%)	2 (66.7%)	4 (80.0%)	2 (100%)			
4.2.2 No respondent	*n* = 22 (44.9%)	8 (57.1%)	4 (40.0%)	7 (50.0%)	3 (100%)			
4.3 21–30%								
All sample	*n* = 26 (7.0%)	17 (21.8%)	9 (13.0%)					
4.3.1 Subsample with collective violence perception	*n* = 3 (4.3%)	2 (12.5%)	1 (7.7%)					
4.3.1.1 Subsample with prediabetes								
4.3.2 No respondent	*n* = 3 (6.1%)	2 (14.3%)	1 (10.0%)					
4.4 31–40%								
All sample	*n* = 13 (3.5%)	8 (10.3%)	5 (7.2%)					
4.4.1 Subsample with collective violence perception	*n* = 3 (4.3%)	2 (12.5%)	1 (7.7%)					
4.4.1.1 Subsample with prediabetes	*n* = 0							
4.4.2 No respondent	*n* = 3 (6.1%)	2 (14.3%)	1 (10.0%)					
4.5 41–50%								
All sample	*n* = 34 (9.2%)					34 (100%)		_
4.5.1 Subsample with collective violence perception	*n* = 2 (5.6%)					2 (100%)		
4.5.1.1 Subsample with prediabetes	*n* = 1 (7.7%)					1 (100%)		
4.5.2 No respondent	*n* = 1 (1.4%)					1 (100%)		
4.6 51–60%								
All sample	*n* = 7 (1.9%)	7 (9.0%)						
4.7 Keep data privacy								
4.7.1 Subsample with collective violence perception	*n* = 8 (11.4%)						8 (100%)	_
4.7.1.1 Subsample with prediabetes	*n* = 0							
4.7.2 No respondent	*n* = 7 (14.3%)						7 (100%)	

* p-Value of all sample and subsample differences between urban center, suburban and urban cluster (Chi-square).

In the second stage, among the screened adolescents, 70 (18.8%) with high scores were contacted for the clinical interview. The response rate was 30, and 61.9% had prediabetes. The adolescents who were screened and did not respond to our call showed more adverse conditions than the other groups, observing that most of them concentrated in suburban grids where 85.7% of the participants had poverty reaching 31–50% of the population (*p* < 0.001). Among them, those who decided to keep their data private were 85.7% female participants and were older than the other groups (*p* < 0.001) (Table 1).

Regarding clinical features, the samples were integrated as shown in Figure 1. There were 12 missing data among samples stratified by poverty in contrast with those stratified by the perception of violence (*n* = 49/*n* = 61, respectively). All the medians and quartiles of the subsamples stratified by violence showed a nutritional status in the eutrophic range, except those with a perception of violence surveyed without prediabetes, who were underweight. In contrast, in the same sample stratified by poverty, only adolescents who lived in contexts where between 35 and 50% of the population was below the poverty line were overweight. In any case, there are no statistical differences between all the subsamples (Table 2B).

**Table 2:** Clinic features by urban poverty sector among the sample.

		**Urban poverty proportion**				** Rural**	** Keep housing data private**	
	**Samples**	**0–18%**	**19–34%**	**35–50%**	**51–70%**			***p*-value Jonckheere-Terpstra Test**
		**Lower**			**Higher**			
	**Median** ** (Q1, Q3)**	**Median** ** (Q1, Q3)**	**Median** ** (Q1, Q3)**	**Median** ** (Q1, Q3)**	**Median** ** (Q1, Q3)**	**Median** ** (Q1, Q3)**	**Median** ** (Q1, Q3)**	
**B. Clical**								
**B1. BMI**								
B1. No perception of collective violence	Median = 20.8 (Q1 = 18.3, Q3 = 23.2)	21.0 (18.0, 23.0)	20.6 (18.8, 23.1)	21.7 (19.0, 23.1)	18.8 (17.7, 22.6)	19.0 (17.3, 23.6)	22.2 (17.3, 25.2)	0.627
B2. Perception of collective violence surveyed	Median = 18.6 (Q1 = 16.5, Q3 = 19.5)			16.5 (16.2, 22.6)	18.5 (18.3, 18.6)			0.266
B3. Perception of collective violence and prediabetes	Median = 21.6 (Q1 = 19.3, Q3 = 24.0)		21.3 (19.4, 21.4)	24.0 (21.5, 25.0)	22.0 (21.8, 22.2)			0.563
B4. Perception of collective violence not surveyed	Median = 21.2 (Q1 = 17.7, Q3 = 23.4)	22.0 (18.4, 24.9)	20.8 (15.6, 26.1)	21.1 (17.8, 23.1)			18.9 (17.7, 22.3)	0.436
Mood's median test (inter-sample diferences)	*p* = 0.171							
**C. Mental health**								
**C.1.1 Social deprivation**								
1.1 No perception of collective violence	Median = 4.0 (Q1 = 1.3, Q3 = 8.0)^*^	6.0 (1.0, 8.3)	3.0 (1.0, 8.0)	4.5 (2.0, 8.0)	5.0 (2.0, 8.0)	4.0 (1.0, 6.0)	4.0 (1.0, 9.0)	0.660
1.2 Perception of collective violence surveyed	Median = 16.0 (Q1 = 8.5, Q3 = 26.5)			10.0 (4.8, 13.0)^*^	26.0 (25.0, 27.0)^*^			0.043
1.3 Perception of collective violence and prediabetes	Median = 18.0 (Q1 = 11.5, Q3 = 27.0)	29.5 (24.0, 35.0)	23.0 (18.0, 28.0)^*^	13.0 (6.5, 25.5)	9.0 (8.0, 10.0)^*^			0.009
1.4 Perception of collective violence not surveyed	Median = 14.0 (Q1 = 9.0, Q3 = 25.0)	15.5 (10.8, 26.0)	9.0 (4.8, 24.5)	13.5 (7.8, 17.8)	23.0 (10.0, 26.5)		21.0 (11.3, 29.5)	0.775
Mood's median test (inter-sample diferences)	*p* < 0.001							
**C.1.2 Contextual adversity**								
2.1. No perception of collective violence	Median = 4.0 (Q1 = 2.0, Q3 = 7.0)^*^	4.0 (1.0, 7.5)	3.0 (2.0, 7.0)	4.0 (2.0, 7.0)	4.0, (2.0, 5.0)	5.0 (4.0, 8.0)	5.0 (2.0, 8.0)	0.442
2.2 Perception of collective violence surveyed	Median = 10.0 (Q1 = 7.5, Q3 = 13.8)			8.0 (4.8, 9.8)^*^	11.5 (10.0, 13.0)^*^			0.015
2.3 Perception of collective violence and prediabetes	Median = 14.0 (Q1 = 9.0, Q3 = 18.0)	16.0 (14.0, 18.0)	18.0 (10.0,18.5)	12.0 (7.5, 19.0)	5.5 (3.0, 8.0)			0.177
2.4 Perception of collective violence not surveyed	Median = 12.0 (Q1 = 8.0, Q3 = 15.0)	11.0 (8.5, 14.5)	11.5 (6.0, 15.8)	12.5 (8.8, 15.3)	12.0 (6.0, 13.5)		13.5 (9.0, 16.0)	0.634
Mood's median test (inter-sample diferences)	*p* < 0.001							
**C.1.3 Violence**								
3.1 No perception of collective violence	Median = 2.0 (Q1 = 0.0, Q3 = 4.0)^*^	2.0 (0.0, 4.0)	1.0 (0.0, 4.0)	2.0 (0.0, 4.0)	2.0 (0.0, 3.0)	4.0 (0.0, 5.0)	3.0 (1.0, 5.0)	0.211
3.2 Perception of collective violence surveyed	Median = 12.0 (Q1 = 9.5, Q3 = 14.8)			10.0 (8.3, 11.8)^*^	13.5 (12.0, 15.0)^*^			0.031
3.3 Perception of collective violence and prediabetes	Median = 14.0 (Q1 = 12.5, Q3 = 17.0)^∧^	17.0 (14.0, 20.0)	16.0 (13.0, 17.0)	13.0 (12.0, 18.0)	10.0 (8.0, 12.0)			0.071
3.4 Perception of collective violence not surveyed	Median = 11.0 (Q1 = 9.0, Q3 = 14.0)	11.5 (9.5, 20.0)	13.0 (8.0, 14.0)	10.0 (8.0, 12.3)	10.0 (8.0, 12.5)		11.0 (9.0, 15.0)	0.206
Mood's median test (inter-sample diferences)	*p* < 0.001							
BMI body mass index								

* ^∧^Statistical differences between pairs (p < 0.05).

In relation to vulnerability, on the one hand, all the aspects explored (social deprivation, contextual adversity, and violence) were statistically different between the subsamples with and without perception of violence, regardless of the diagnosis of prediabetes, showing lower scores among those who did not perceive vulnerability (*p* < 0.001). It is relevant that in the same aspect of violence, the subsample of prediabetes reported higher scores and showed statistical differences with non-respondents (*p* = 0.006). On the other hand, in terms of poverty, the most affected groups (35–50% and 51–70% of their population below the poverty line) concentrate the majority and the differences between them are statistically significant, with adolescents in the highest poverty group having the poor perception of vulnerability (*p* < 0.043) (Table 2C).

As for the comparative analysis among the groups according to the use of health services and diagnosis, it shows that, although there were no statistical differences, service users lived in neighborhoods with a lower proportion of poverty than the newly diagnosed, who were more disadvantaged. In total, 94.4 and 78.9% of participants in the school subsamples with and without prediabetes, respectively, had some healthcare coverage, and 16.7 and 47.4% of their families were themselves receiving assistance from a social program (Seguro Popular), including health services, whereas none in the health services group were under this protection. The group of adolescents with T2DM stood out among all other groups for showing the highest social wellbeing, especially those who did not report violence exposure, 75% of whom lived in contexts of lower poverty, and had the greatest access to health services, even, all of them accessing private services (Table 3).

**Table 3:** Comparative analysis of sociodemographic and clinical features of adolescents with prediabetes.

	**Health services users**					**All newly diagnosed participants** ** in school (hidden)**			**School participants** ** without prediabetes**			***p*-Value Chi-square and Kruskall Wallis tests**
	**Type 2 diabetes**		**Prediabetes**		**Health services users with prediabetes**	**Prediabetes**						
	**Violence exposure**		**Violence exposure**			**Violence exposure**			**Violence exposure**			
	**With**	**Without**	**With**	**Without**	**Total**	**With**	**Without**	**Total**	**With**	**Without**	**Total**	
	***n* = 5**	***n* = 4**	***n* = 1**	***n* = 3**	***n* = 4**	***n* = 12**	***n* = 6**	***n* = 18**	***n* = 16**	***n* = 3**	***n* = 19**	
Edad	15 (1, 17)	13 (11.3, 16.3)	15	10.0 (9.0, 13.0)	11.5 (9.3, 14.5)	13.0 (13.0, 14.0)	14.0 (13.0, 15.0)	13.0 (13.0, 14.0)	13.0 (13.0, 14.0)	14.0 (13.0, 17.0)	13.0 (13.0, 14.0)	0.341
Sexo (female)	3 (60%)	1 (25%)	1 (100%)	2 (66.7%)	3 (75%)	6 (50%)	3 (50%)	9 (50%)	7 (43.8%)	1 (33.3%)	8 (42.1%)	0.484
**A. Urban poverty proportion by residence**												
0–18%		3 (75%)				1 (8.3%)	2 (33.3%)	3 (16.7%)		1 (33.3%)	1 (5.3%)	0.375
19–34%	2 (40%)	1 (25%)		3 (100%)	3 (75%)	4 (33.3%)	4 (66.7%)	8 (44.4%)	4 (25%)	1 (33.3%)	5 (26.3%)	
35–50%	3 (60%)		1 (100%)		1 (25%)	5 (41.7%)		5 (27.8%)	5 (31.3%)	1 (33.3%)	6 (31.6%)	
51–70%						1 (8.3%)		1 (5.6%)	6 (37.5%)		6 (31.6%)	
Rural						1 (8.3%)		1 (5.6%)	1 (6.3%)			
Unknown adress (keep data privacy)												
**B. Health care services profiles**												
B1. Coverage of health services	5 (100%)	4 (100%)	1 (100%)	3 (100%)	4 (100%)	11 (91.7%)	6 (100%)	17 (94.4%)	12 (75%)	3 (100%)	15 (78.9%)	0.509
Missing						1 (8.3%)		1 (5.6%)	3 (18.8%)		3 (15.8%)	
Healthcare provider												0.096
B1.1 Public assistence healthcare services “Seguro Popular”	4 (80%)	2 (50%)				6 (50%)		6 (33.3%)	7 (43.8%)	2 (66.7%)	9 (47.4%)	
**% Under prospera social program**	1 (20%)	3 (75%)				3 (25%)		3 (16.7%)	7 (43.8%)	2 (66.7%)	9 (47.4%)	0.061
B1.2 Social security health care services												
B1.2a No goverment workforce sector “IMSS”	1 (20%)	1 (25%)	1 (100%)	3 (100%)	4 (100%)	4 (33.3%)	3 (50%)	7 (38.9%)	4 (25.0%)		4 (21.1%)	
B1.2b Goverment workforce sector “ISSSTE”		1 (25%)				1 (8.3%)	3 (50%)	4 (22.2%)	1 (6.3%)		1 (5.3%)	
B2. Diabetes care services use by level												
Primary care services (basic services TI)	1 (20%)											
Primary care services (advanced services TIII)				2 (66.6%)	2 (50%)							
Primary care (specialized services on diabtes UNEME)	3 (60%)											
Specialized services			1 (100%)		1 (25%)							
Private specialized services	1 (20%)	4 (100%)		1 (33.3%)	1 (25%)							
**C. Clinical profiles**												
**Previous diagnosis**	5 (100%)	4 (100%)	1 (100%)	1 (33.3%)	2 (50%)							
Diabetes/pre-diabetes age of diagnosis	14.0 (11.0, 14.5)	8.5 (2.0, 9.8)	14	11.0 (9.0, 13.0)	12.0 (9.5, 13.8)	13.0 (13.0, 14.0)	14.0 (13.0, 15.0)	13.0 (13.0, 14.0)			_	0.073
**C1. Endocrine markers**												
**HbA1c (%)**	7.8 (6.3, 11.9)	5.8 (5.7, 6.4)	5.6	5.2 (4.7, 5.7)	5.6 (4.7, 5.7)^*^	5.8 (5.8, 5.9)	5.85 (5.8, 5.9)	5.8 (5.8, 5.9)^*^∧	5.4 (5.2, 5.5)	5.6 (5.4, 5.6)	5.4 (5.3, 5.6)^∧^	0.001
BMI	24.7 (19.2, 34.8)	24.5 (21.3, 31.0)	40.5	31.8 (22.3, 40.0)	35.9 (24.7, 40.4)^*^∧	22.2 (19.3, 24.6)	21.5 (19.8, 41.0)	21.8 (19.4, 25.2)^*^	19.1 (17.0, 23.2)	27.6 (20.1, 35.0)	19.6 (17.9, 24.9)^∧^	0.031
Missing	_	_		_	_	3 (25%)	1 (16.7%)	4 (22.2%)	4 (25.0%)	1 (33.3%)	5 (26.3%)	
HOMA-IR	3.3 (0.8, 16.3)	1.6 (0.4, 2.3)	4.9	8.9 (3.9, 13.9)	4.9 (3.9, 13.9)^*^∧	2.4 (1.1, 3.9)	1.8 (1.0, 2.8)	2.2 (1.2, 3.0)^*^	1.5 (1.1, 2.5)	2.8	1.6 (1.2, 2.6)^∧^	0.025
Missing	1 (20%)	_		1 (33%)	1 (25%)	1 (8.3%)	_	1 (5.6%)	_	2 (66.7%)	2 (10.6%)	
Cortisol	11.3 (8.6, 12.2)	9.4 (8.6, 11.0)	11.8	11.2	11.5 (11.2, 11.5)	8.6 (5.8, 13.6)	7.0 (6.8, 12.9)	8.6 (6.8, 13.3)	7.8 (6.2, 10.3)	13.2	8.1 (6.3, 11.1)	0.451
Missing	1 (20%)	_		2 (66.6%)	2 (50%)	_	1 (16.7%)	2 (11.1%)	_	2 (66.7%)	2 (10.6%)	
Comparartive groups in shading colums												

* ^∧^Statistical differences between pairs (p < 0.05).

Regarding endocrine markers, newly diagnosed adolescents had the worst metabolic control (*p* = 0.001). Being overweight, obesity (*p* = 0.031), and insulin resistance (*p* = 0.025) affect adolescents with prediabetes in health services to a greater extent. Likewise, analyzing all groups with exposure to violence, their cortisol, and HOMA-IR medians were higher than those of their peers in the same group, although, they did not reach statistical significance, which could be related to the numerous missing values (up to 66.3% in the very small groups).

## Discussion

This brief report is a secondary analysis of data from another main study aimed to associate exposure to collective violence and pre/diabetes. An unexpected proportion of adolescents with prediabetes, who had not been diagnosed until enrollment into the study and remained hidden from the health system, was observed. The report presents a quantification and description of this phenomenon, highlighting the social determinants that influence the lack of diagnosis, as well as comparing access to health services in different demographic contexts and exploring clinical profiles.

There are different studies about the prevalence of prediabetes in Mexico; although there are no representative samples, a high proportion of the undiagnosed population among adolescents up to 14.3% has been described (3, 5–7). However, our approach to disadvantaged contexts could be identified, after screening for vulnerability, a target subsample highly affected by prediabetes, reaching 61.9%, probably because of the inclusion of locations where social determinants linked to the onset of the disease converge (17, 33). It is important to note that our estimate of this proportion was obtained by measuring 30% of the sample of the adolescents most affected by social adversity, whose risks are increased by their vulnerability (9, 34–36). Moreover, this proportion includes only those affected by collective violence, who appear to be more at risk of being metabolically and mentally compromised, and we did not consider peers without this perception. Therefore, it is far from relating to the national epidemiological pattern, since it neither represents it nor is it extrapolated to the general pediatric population.

The most affected adolescents were those living in highly excluded areas where 35–70% of the population lives below the poverty line. The high proportion of prediabetes could have responded to the fact that these adolescents were affected, by low household income and lack of food security that prevent access to a healthy diet. Also, a lack of public safety reduces healthy spaces and favors a sedentary lifestyle (36, 37). Moreover, there is a consistent finding of more affected endocrine markers in those with exposure to violence compared to their peers without this exposure. In fact, these social determinants cause severe psychological stress that could promote a pro-inflammatory state leading to metabolic impairment, including prediabetes (38–41).

Despite enrollment in healthcare services and social programs, there were significant barriers related to availability and accessibility to health services, which were promoted by social conditions. Regarding the barriers to access, affiliation is the first step to achieving effective access to such services, but it is not found to be enough; effective and efficient care to health problems resolution is also required (42). Most of the participants had some affiliation, but the density of health facilities, their household, geographic- (road communication, transport), financial- and healthcare services organizational-related barriers, limited the reach of the health system to registry and diagnosis (43). All these conditions reinforce the vulnerability of this population to remain hidden.

The poor social conditions of non-respondents were highlighted, it may be that the harsh social exclusion reinforces the barriers to health services (44). Despite the intentional search for this population, efforts were insufficient to reach them. In addition, their likely subjective status as a hidden population may have contributed to the non-response. Most of the women who did not respond kept their data private and did not go on to the second phase of the study, resulting in gender inequality; therefore, strengthening gender is a social determinant that plays an important role in adolescent health coverage (45). This might have been because they perceived greater vulnerability and avoided making themselves visible in relation to their conditions as a hidden population (46).

Among the newly diagnosed adolescents, about 50% had an affiliation with “Seguro Popular,” the public assistance health services, which reaches the most excluded localities offering basic services as “T1 units.” In one report, “Seguro Popular” had no effect on the use of preventive care, such as screening for chronic diseases of high prevalence in the adult population, which is not considered a public policy for adolescents (47). But in another report, the use of these preventive services was even lower among those affiliated with “Seguro Popular” compared to those affiliated with worker's services (48). It is to this sector that the other 50% of the participants belong and have the most frequent affiliation among the subsample of health service users. Indeed, those who have this kind of social security show better social conditions.

However, a proportion of Seguro Popular users (about 11.5%) were known to have affiliations with other public health service providers and about 46.4% used private services in 2018 (49). This fact is linked to barriers related to the availability of health services for adolescents who had an affiliation with worker's services and who lived in the suburban grid and urban clusters, but who did not have health facilities corresponding to this main affiliation. Therefore, at best, they used the basic public healthcare services offered in the “T1 units,” which implied the same social conditions that favored the lack of diagnosis. The resulting underutilization of services prevented the assistance of the hard-to-reach excluded population (45).

Effective access to healthcare and the vulnerability of individuals have been described as influencing factors for the diagnosis of glucose impairment and early detection (9, 50). The users of health services in the study showed the best social conditions among the whole sample, and it was more evident in those who did not report exposure to violence and who showed the least poverty among all groups, despite the non-significant differences due to the small subsamples. These could have been favored by protective social factors in this privileged sector (51). Among these same users group highlight that wellbeing is not only due to income and security but also access to health services, even reporting the use of specialized and properly qualified private services among 100% of adolescents in health services without exposure to violence.

In contrast, in this study, the included screened participants showed the worst social conditions, which was consistent with their report of vulnerability showing high scores on all aspects of adversity explored, compared to their unscreened scholar peers (*p* > 0.001). Chronic exposure to severe stressors has been suggested to interfere with the endocrine and metabolic balance of individuals (38, 52). Thus, all newly diagnosed adolescents showed a pattern of increased cortisol and insulin resistance compared with peers without exposure. According to the literature, it has been reported that patients with glucose disturbances, mainly diabetes, show an alteration of multisystemic responses to stress (40, 41, 53–55). The same pattern is seen among participants with T2DM with and without exposure to violence.

Regarding metabolic outcomes, although the newly diagnosed adolescents had the worst HbA1c of the entire sample of patients with prediabetes, their insulin resistance and BMI were below the median of healthcare users. Regarding HbA1c, it is plausible that adolescent users of health services, due to favorable conditions, achieve glycemic control. However, overweight and obesity seem to remain despite their management and treatment resources, it appears to be the same challenge for other user populations of health services (56, 57).

Notwithstanding the well-studied direct association between BMI and insulin resistance, the newly diagnosed participants in our study showed eutrophic nutritional status that is related to a median insulin resistance slightly below the cut-off point of the pediatric consensus value (58). It is important in a way that many studies on the occurrence of prediabetes have been conducted in overweight and obese samples, even diagnostic suspicion has been established among adolescents with these comorbid conditions (3). Additional in-depth studies, based on screening for prediabetes in eutrophic adolescents with other related conditions, such as social adversity, may lead to broadening our understanding of the importance of social determinants in the onset of this complex disease.

## Conclusion

The results reported an unexpectedly high proportion of adolescents with prediabetes who live in contexts under conditions of very harsh social adversity. Differences among groups in poverty, exposure to violence, and access to health services suggested that these determinants affected the process of perceiving vulnerability and the health of these adolescents, imposed barriers to access to health services, and promoted conditions that keep them hidden from the health system. The reported wide coverage of public health services and social programs, however, does not seem to be able to improve these social determinants, mainly violence. Health promotion strategies aimed at mitigating the impact of conditions of poverty and collective violence are needed to improve the diagnosis, management, and control of prediabetes.

## Limitations

The limitations of the study are related to the selection biases outlined in the corresponding section. It is important to note that the study has external validity limitations, as it presents information from a population in harsh social adversity. Their exposures, mainly to collective violence, are not comparable to other contexts or clinical populations widely studied and reported in the world literature. Moreover, their hidden condition is favored by the limited access to healthcare services to the most excluded population. It is possible that the found proportion of prediabetes was related to the over-representation of adolescents with risk accumulation for the early onset of the disease.

## Data availability statement

The datasets for this study can be found in the Supplementary material (Data Sheets 1, 2). Further inquiries can be directed to the corresponding author.

## Ethics statement

This research was approved by the Institutional Ethics Committee of National Institute of Pediatrics no. 021/2018, registered at the Office for Human Research Protection of the NIH (http://ohrp.cit.nih.gov/search/search.aspx) with numbers IRB00008064 and IRB00008065. Approval is available upon request. Written informed consent to participate in this study was provided by the participants' legal guardian/next of kin.

## Author contributions

DH-M: conception and design of the work, acquisition, analysis, and interpretation of data and drafting the work, provide approval for publication of the content, and accountable for all aspects of the work in ensuring that questions related to the accuracy or integrity of any part of the work are appropriately investigated and resolved. EC-E, ME-A, and AM-T: acquisition, analysis, and interpretation of data for the work and drafting of the work, provide approval for publication of the content, and accountable for all aspects of the work in ensuring that questions related to the accuracy or integrity of any part of the work are appropriately investigated and resolved. All authors contributed to the article and approved the submitted version.

## Funding

This work was supported in part by the National Council for Science and Technology (CONACyT) project 221695 and in other by the National Institute of Pediatrics (Instituto Nacional de Pediatría) project 021/2018.

## Conflict of interest

The authors declare that the research was conducted in the absence of any commercial or financial relationships that could be construed as a potential conflict of interest.

## Publisher's note

All claims expressed in this article are solely those of the authors and do not necessarily represent those of their affiliated organizations, or those of the publisher, the editors and the reviewers. Any product that may be evaluated in this article, or claim that may be made by its manufacturer, is not guaranteed or endorsed by the publisher.
