# Supplementary material for: Undiagnosed prediabetes in Mexican adolescents under poverty in contexts affected by collective violence: A clinical comparison among health services users and hidden population
Source: Front Nutr. 2022 Nov 21;9:1007781. doi: 10.3389/fnut.2022.1007781 (PMC9720165; doi:10.3389/fnut.2022.1007781)
Supplement: Supplementary file 2 [file Data_Sheet_1.pdf]

Subject	Dx	Violence	Deprivation	Contextual	Context	Poberty	ProportionHS	Age	Sex	BMI	Phase
1	6	0	0	1				14	1	24.56	2
2	6	1	9	16	2	2	1	13	2	15.24	2
3	6	1	3	5	2	2	3	13	1	24.30	2
4	6	20	10	12	2	1	2	14	1	16.82	2
5	6	12	17	13	2	3	1	13	1	26.35	2
6	6	8	16	9	1	1	4	13	2	16.41	2
7	6	9	17	16	2	3	1	15	1	21.73	2
8	6	6	4	4	2	3	1	14	2	21.63	2
9	6	20			9	9	9	15	1		2
10	6	3	10	1	2	3	2	13	1	22.51	2
11	6	8	11	13	2	3	1	13	2	21.75	2
12	6	11	34	16	9	9	9	14	1	23.34	2
13	6	2	23	4	2	2	1	14	2	18.22	2
14	6	4	9	5	2	2	3	14	2	18.37	2
15	6	0	3	0	1	3	99	13	2	22.31	2
16	6	2		13				13	1	19.47	2
17	6				2	2	2	13	2	17.78	2
18	6	0	4	8	1	2	3	13	1	21.22	2
19	6	0	0	0	2	2	2	14	1		2
20	6	2		13	2	3	1	13	1	19.47	2
21	6	0	6	0	1	2	4	13	1	23.88	2
22	6				9	9	9	13	2	22.22	2
23	6	3	2	0	1	2	1	13	2		2
24	6	2	3	6	1	1	1	13	2	22.58	2
25	6	2	4	8	2	1	2	13	1	19.22	2
26	6	0	1	8	2	2	3	13	2	23.18	2
27	6	2	1	7				14	2	23.98	2
28	6	4	6	8				13	2	24.22	2
29	6	2	6	17	2	3	1	14	1	18.03	2
30	6	3	3	12	1	1	2	13	1	24.13	2
31	6	0	2	1				13	2	16.65	2
32	6	1	1	11	2	3	1	13	1	14.85	2
33	6	1	1	5	2	3	2	14	1	24.97	2
34	6	1			2	3	1	13	2	16.65	2
35	6	0	5	20	4	5	5	14	2	18.94	2
36	6				2	3	1	13	2	27.12	2
37	6	0	1	5	2	4	2	14	2	32.03	2
38	6	5	2	3	2	3	2	13	2	24.44	2
39	6	2	4	8	2	3	2	13	2	22.89	2
40	6	0	3	11	1	2	1	13	1	23.44	2
41	6	2	8	4	2	4	2	13	1		2
42	6	1	3	3	2	4	2	13	1	17.90	2
43	6	1	4	6	9	9	9	14	1	13.74	2
44	6	1	4	5	9	9	9	13	1	16.44	2
45	6	3	1	3	2	3	1	13	1	17.10	2
46	6	2	1	1	2	3	2	13	1	19.19	2
47	6	4	16	3	2	3	1	13	2	19.83	2
48	6	0	0	0	2	4	2	14	2		2
49	6	0	0	1	2	3	2	15	2	22.20	2
50	6	3	5		2	3	1	13	2	20.08	2
51	6	0	0	0	2	3	2	13	2	25.71	2
52	6	3	9	9	2	3	2	13	2	23.15	2
53	6	7	2	5	2	3	2	14	2	15.81	2
54	6	2	2	4	2	3	1	14	2	27.77	2
55	6	6	5	5	2	3	1	13	1	22.52	2
56	6	5	14	7	9	9	9	15	1	18.29	2
57	6	5	10	7	9	9	9	13	1		2
58	6	0	3	0				14	1	18.97	2
59	6	4	2	3	2	4	2	13	2	18.52	2
60	6	4	2		2	3	1	14	2	20.96	2
61	6	0	0	0	2	3	1	14	2	18.42	2
62	6	0	19	3	1	1	2	13	2	18.67	2
63	6	2	8	4	2	3	2	14	1	24.09	2
64	6	11	15	16	2	3	1	13	1		2
65	6	0	0	2				13	1		2
66	6	3	4	12	1	2	1	13	1	19.23	2
67	6	3	7	6	2	3	1	14	1	17.78	2
68	6	4	8	9	2	3	2	13	1		2
69	6	1	0	3	1	2	2	13	1	23.44	2
70	6	8	10	6	1	2	1	14	1	15.63	2
71	6	5	9	6	1	3	99	13	1	19.92	2
72	6	2	4	4	1	1	2	14	1	15.63	2
73	6	2	8		2	2	2	13	1	19.53	2
74	6	3	17	5	2	2	99	13	1	23.44	2
75	6	2	13	4	2	3	99	13	1	23.44	2
76	6	5	0	7	1	2	2	13	1	20.61	2
77	6	0	6	5	2	3	2	13	1	32.84	2
78	6				2	4	2	13	1	18.75	2
79	6	0	0	0	9	9	9	13	1	17.15	2
80	6	8	3	5	2	3	1	13	1	18.30	2
81	6	1	1	2	9	9	9	13	1	25.71	2
82	6	1	6	1	2	3	1	13	1	22.83	2
83	6	13	33	11	2	3	2	14	1	20.57	2
84	6	1	6	2	9	9	9	14	1	22.20	2
85	6	1	2	2	9	9	9	14	1	17.44	2
86	6	9	36	15	2	3	1	13	1	23.11	2
87	6	1	0	0	2	3	1	13	1	19.83	2
88	6	2	7	3	2	4	2	13	1	23.11	2
89	6	8	8	8	2	3	2	13	1		2
90	6	2	5	2	2	3	2	13	1	20.41	2

91	6	4	3	3	2	3	2	14	1	18.92	2
92	6	8	12	9	1	1	2	14	1	23.88	2
93	6	9	13	9	9	9	9	14	1	19.36	2
94	6	10	14	20	2	3	2	14	1	23.11	2
95	6	3	2	3	9	9	9	14	1	24.61	2
96	6	0	4	3	2	3	2	13	1		2
97	6	0	6	4	2	3	1	13	1	18.49	2
98	6	1			2	3	1	13	1		2
99	6	8	30	12	2	4	2	13	1		2
100	6	2	12	11	2	3	2	13	1		2
101	6	6	20	10	2	3	1	13	1	19.53	2
102	6	0	5	10	2	3	1	13	1		2
103	6	1	12	7	2	3	1	13	1	25.00	2
104	6	8	9	10	2	3	2	14	1	17.31	2
105	6	4	11	4	2	3	2	14	1	22.51	2
106	6	0	6	1	1	1	4	14	1	15.06	2
107	6	2	7	5	2	4	1	15	1	18.75	2
108	6	3	9	8	9	9	9	13	1		2
109	6	5	12	9	9	9	9	13	1	22.67	2
110	6	8	28	16	9	9	9	13	1		2
111	6	0	0	2	2	3	2	13	1		2
112	6	0	1	3	2	3	2	14	1	23.11	2
113	6	2	12	2	2	3	2	14	1		2
114	6	4	3	8	2	4	2	14	1	26.22	2
115	6	0	13	1	3	3	1	14	1		2
116	6	6	2	4	2	3	99	14	1		2
117	6				9	9	9	13	2	26.31	2
118	6	6	10	8	2	3	2	13	2	24.31	2
119	6	2	2	1	2	3	1	14	2	23.39	2
120	6	0	2	0	2	3	1	13	2	12.34	2
121	6	1	7	7	2	3	2	14	2	17.94	2
122	6	0	2	5				13	2	22.22	2
123	6	0	0	1				13	2		2
124	6	13	13	14	1	1	2	13	2	19.76	2
125	6	13	28	16				15	2	17.31	2
126	6	0	5	5	2	3	2	13	2	15.43	2
127	6	10	3	13	2	3	2	14	2	17.33	2
128	6	2	3	5				14	2	22.98	2
129	6	2	3	2	2	3	2	14	2	22.37	2
130	6	4	13	7	1	1	4	13	2		2
131	6	0	2	8				14	2	18.14	2
132	6	1	10	6	2	1	2	13	2	19.53	2
133	6	2	10	5	1	1	3	13	2	21.03	2
134	6	5	0	2	1	2	4	13	2	22.58	2
135	6	4	7	9	1	1	2	14	2	19.53	2
136	6		12	3	1	2	1	14	2	19.61	2
137	6	0	4	0	1	1	6	14	2		2
138	6	0	2	8	2	2	2	13	2	23.44	2
139	6	10	3	8	2	1	3	14	2	19.53	2
140	6	0	0	2	1	1	1	13	2	25.99	2
141	6	3	4		2	3	2	13	2	21.56	2
142	6	4	10					12	2	21.39	2
143	6	0	2	0				13	2		2
144	6	3	7	7	2	3	1	14	2	19.10	2
145	6	1	1	3	2	3	2	15	2		2
146	6	4	3	4	2	3	2	14	2		2
147	6	5	6	1	2	4	2	14	2	16.00	2
148	6	0	2	0	2	3	2	13	2	17.51	2
149	6	3	3	9				13	2	22.77	2
150	6	6	5	16	2	3	2	14	2	21.74	2
151	6	0	0	0				14	2		2
152	6	0	0	5	9	9	9	14	2	22.43	2
153	6	15	23	15	2	4	2	13	2	12.86	2
154	6	2	0	0				13	2		2
155	6	5	8	10	2	3	2	14	2	20.85	2
156	6	0	8	2	9	9	9	13	2		2
157	6	4	13	6	9	9	9	12	2		2
158	6	0	0	4	2	3	1	13	2	25.83	2
159	6	3	1	0	2	2	1	13	2	14.80	2
160	6		13		2	3	2	14	2	18.84	2
161	6	5	1	5	9	9	9	16	2	22.66	2
162	6	10	21	15	1	2	1	14	2	14.88	2
163	6	11	6	9	9	9	9	14	2		2
164	6	0	6	3	2	3	2	14	1	25.59	2
165	6	6	8	15	2	3	1	15	1	22.21	2
166	6				1	1	2	13	1		2
167	6								1		2
168	6				2	1	1	14	2		2
169	6		2	0	2	1	2	13	2	12.03	2
170	6	4	6	6	1	2	1	13	1		2
171	6	15	25	12	9	9	9	14	1	17.54	2
172	6	7	7	9	9	9	9	13	1		2
173	6	3	1	4	1	1	2	13	1	23.44	2
174	6	4	14	20	1	1	2	14	1	22.72	2
175	6	0	0		1	1	2	13	2	23.46	2
176	6	12	8	13	1	2	2	14	2	27.10	2
177	6	8	15	7	2	1	2	13	2	21.05	2
178	6	4		4	1	1	2	14	1		2
179	6	2	8	15	1	1	1	13	2	16.64	2
180	6	7	8	5	1	2	2	13	1	16.41	2
181	6	8	4	5	2	2	2	13	2	21.40	2
182	6	3	8	3	1	1	3	14	2	14.53	2

183	6	20	29	14	2	1	1	13	2		2
184	6	10	11	11	1	1	2	13	2	27.36	2
185	6	12	20	9	2	3	1	13	1	17.98	2
186	6	3	2	6	1	1	3	13	2	22.21	2
187	6	16	33	15	1	1	4		1	26.31	2
188	6	14	40	11	2	2	99	14	1	20.12	2
189	6	4	8	6	1	1	6	13	2	22.35	2
190	6	2	5	0	1	1	6	13	2	17.54	2
191	6	7	10	6	1	2	1	14	1	20.57	2
192	6	3	5	15	9	9	9	14	2		2
193	6	5	9	11	2	2	2	13	2	23.44	2
194	6	6	15	11	1	1	3	13	1	23.01	2
195	6	20	17		1	1	3	13	2	23.44	2
196	6	3	8	11	1	3	1	13	1	21.93	2
197	6	2	24	5	1	1	3	14	1		2
198	6	0	0	0	1	1	3	13	2	14.95	2
199	6	3	3	4	9	9	9		2	14.19	2
200	6	5	7	16	2	1	3	14	2		2
201	6	6	16	19	1	1	2		2	21.91	2
202	6	8	8	10	2	99	99	13	2	23.44	2
203	6	20	2	11	1	1	2	13	1		2
204	6	10	25	15	1	1	1	13	2	17.98	2
205	6	10	17	15	9	9	9		1	18.37	2
206	6	0	25	5	1	1	2	13	1	24.46	2
207	6	2	7	9	1	1	6	14	1	21.00	2
208	6	0	6	2	1	1	4	14	2	25.78	2
209	6	8	8	12	1	2	4	13	2	15.62	2
210	6				1	2	1	13	2	19.53	2
211	6	0	0	1	2	2	3	13	1	19.22	2
212	6	2	1	0	1	1	2	13	1	21.26	2
213	6	2	1	3	1	1	2	14	1		2
214	6	5	16	1	2	2	99	13	2	19.83	2
215	6	0	0	2	1	2	2	13	2	14.53	2
216	6	2	3	9	2	2	3	13	2	17.51	2
217	6	3	0	3	1	1	6	12	1	15.63	2
218	6	0	1	1	1	2	2	13	1	19.57	2
219	6	0	7	2	2	2	2	14	1	19.22	2
220	6	14	14	18	1	2	3	14	1		2
221	6	0	2	2	1	2	4	13	1	20.76	2
222	6	2	5	8	1	1	3	14	2	17.96	2
223	6	2		2	1	1	6	13	2	23.03	2
224	6	4	8	1	1	2	3	13	1	22.86	2
225	6	14	5	6	2	2	2	13	1		2
226	6	0	0	0	1	1	4	13	1	20.00	2
227	6	0	1	12	1	1	4		1	22.83	2
228	6	5	25	7	2	2	1	13	2	22.15	2
229	6	4	2	3	1	2	1	14	1	24.46	2
230	6	0	0	3	1	2	2	13	1	23.90	2
231	6	0	0	1	1	1	3	13	1	15.82	2
232	6	13	23	7	1	1	2	13	2	22.86	2
233	6	0	0		1	2	2	14	1	17.78	2
234	6	2	3	7	2	2	2	13	1	18.22	2
235	6	4	11	9	1	2	1	13	2		2
236	6	2	2	4	1	1	3	14	2	22.49	2
237	6	4	4	3	1	2	2	13	1		2
238	6	4	15	5				15	2		2
239	6	8	23	7				13	1	22.35	2
240	6	0	7	13	2	3	2	14	1	23.11	2
241	6	4	25	8	1	1	2	13	1	21.45	2
242	6	0	9	0	1	1	2	13	1	19.92	2
243	6	1	7	0	1	1	2	13	1	20.41	2
244	6	0	0		1	2	2	14	2	21.51	2
245	6	0	1	7	1	2	2	13	1	19.53	2
246	6	0	2	1	1	1	4	13	1	18.22	2
247	6	1	5		1	2	2	13	1	22.35	2
248	6	0	0	0	9	9	9	13	2	17.58	2
249	6	4	1	3	1	2	4	14	1	21.26	2
250	6	5	6		2	2	1	13	2	17.58	2
251	6	4	9	5	1	1	2	13	2	19.22	2
252	6	0	6	2	1	1	6	13	2	16.02	2
253	6	0	1	1	2	2	2	14	1	20.62	2
254	6	4	7	5	2	2	3	13	2	16.02	2
255	6	1	6	0	1	1	2	14	1	18.37	2
256	6	0	5	15	1	1	2	13	2	18.22	2
257	6	4	1	3	1	1	3	13	2	23.19	2
258	6	4	6		1	1	2	13	2	37.76	2
259	6	1	3	2	2	2	99	13	1	26.64	2
260	6	1	0	4	1	2	1		1	23.11	2
261	6	1	4	0	1	2	2	13	2	22.60	2
262	6	4	2	14	9	9	9		1	26.90	2
263	6	0	0	0	1	1	2	13	2	24.61	2
264	6	1	7	1	1	1	2	13	2	24.69	2
265	6	10	29	17	1	1	2	13	1	25.20	2
266	6	7	18		1	1	3	13	2	15.06	2
267	6	0	7	6	1	1	1	13	1	24.22	2
268	6	0	1	0	1	1	3	13	2	22.04	2
269	6	5	13	6	1	1	2	13	2	20.00	2
270	6	1	4	2	1	2	2	13	2	21.09	2
271	6	0	0	0	2	2	2	13	1	22.22	2
272	6	1	8	5	1	2	2	13	2	25.51	2
273	6	1	2	2	1	1	3	13	1	22.48	2
274	6	0	1	0	1	1	2	15	1	17.98	2

275	6	1	2	0	2	3	2	13	1	19.05	2
276	6	0	0	3	2	2	2	13	2	19.92	2
277	6	5	0	4	2	1	1	13	2		2
278	6	6	6	10	1	1	2	13	2		2
279	6	2	8	6	1	1	3	13	1	24.09	2
280	6	12	17	16				13	1		2
281	6	7	10	15				13	1		2
282	6	20	39	20				14	1	23.22	2
283	6	4	3	7	3	3	2	13	1		2
284	6	16	7	12	3	3	2	13	1		2
285	6	5	12	6				13	2	25.80	2
286	6	12	9	12				13	1	25.90	2
287	6	1	1	0				13	2		2
288	6	6	24	9				13	1		2
289	6	3	2	4				13	1	27.70	2
290	6	3	2	4	3	4	2	13	1		2
291	6	11		0				13	2		2
292	6	3	3	4				14	1	19.50	2
293	6	4	2	8	4	5	5	13	2	23.60	2
294	6	3	4	4				13	2		2
295	6	5	3						1		2
296	6	9	17	14				13	2		2
297	6	1	19	5				13	2		2
298	6	2	1	1				13	1		2
299	6	2	3	3				13	1		2
300	6	1	3	4				13	1		2
301	6	3	11	1				13	1		2
302	6	11	12	10				13	2		2
303	6	2	6	11				14	2		2
304	6	4	4	3				13	1		2
305	6	11	10	8				13	1		2
306	6	20	27	10				13	2		2
307	6	5	15	9				13	1		2
308	6							14	1		2
309	6	10	10	6	3	4	2	13	2		2
310	6	14	13	0	3	3	2		2		2
311	6	3	9	4	3	4	2		1		2
312	6	2	4	10	9	9	9		1		2
313	6	5	4	5	4	5	5		1		2
314	6							13	1		2
315	6	3	1	3					2		2
316	6	10	3	6					2		2
317	6		3					13	2		2
318	6	0	5		3	4	2	13	1		2
319	6	0	4	3				13	1		2
320	6	6	6	8				13	1		2
321	6	4	6	8	4	5	5	13	2		2
322	6	0	0	3	3	3	2	13	1		2
323	6	3	16	4	3	4	2	13	1		2
324	6	0	0	3				13	2		2
325	6	1	2	3				13	2		2
326	6	5	2	7				13	1		2
327	6	2	8	4				14	1		2
328	6	10	10	5				13	1		2
329	6	0	4	4				13	1		2
330	6	2	3	4				13	1		2
331	6	0	7	2				14	1		2
332	6	5	6	5	3	3	2	13	1		2
333	6	4	3	9					2		2
334	6	0	8	15					1		2
335	6	5	10	3	9	9	9		1		2
336	6	0	0	0	3	4	2		2		2
337	6	0	9	6	3	4	2		2		2
338	6	2	6	2	3	4	2		1		2
339	6	5	5	5					1		2
340	6	1	4	2					1		2
341	6	7	11	10					1		2
342	6	2	0	4	4	5	5	13	1		2
343	6	6	9	5	3	4	2		1		2
344	6	3		7				13	1		2
345	6	0	0	0	3	3	2	13	2		2
346	6	3	8	4				14	1		2
347	6	2	5	2	3	4	2	13	1		2
348	6	2	1	1				13	1		2
349	6	4	18	8				13	1		2
350	6	5	15	12				13	1		2
351	6	7	6	5	4	5	5	13	2		2
352	6	0	1	2				13	1		2
353	6	3	3	5	3	4	2	13	2		2
354	6	0	0	0	3	3	2	13	1		2
355	6	0	0	4				13	1		2
356	6	0	2	2				14	2		2
357	6	4	18	5				13	1		2
358	6	5	3	4	3	3	2	13	1		2
359	6	5	34	13				13	2		2
360	6	1	6	3				14	2		2
361	6	0	1	1	9	9	9	13	1		2
362	6	5	7	5	3	3	2	13	1		2
363	6	2	4	0	3	3	2	13	2		2
364	6	0	1	1	4	5	5	14	2		2
365	6	1	6	3				13	2		2
366	6				4	5	5	13	1	19.05	2

367	6				4	5	5	12	2	16.23	2
368	6				4	5	5	12	2	17.29	2
369	6				3	4	2	15	2	16.55	2
370	6				4	5	5	16	2	18.03	2
371	6				3	4	2	15	1	21.64	2
372	6				4	5	5	15	1	18.95	2
373	6				3	4	2	15	1	28.45	2
374	6				4	5	5	12	2	16.65	2
375	6				3	4	2	15	2	24.98	2
376	6				3	4	2	14	2	20.53	2
377	6				4	5	5	15	1	18.22	2
378	6				3	3	2	15	1	23.16	2
379	6				4	5	5	13	1	17.12	2
380	6				3	4	2	14	2	18.10	2
381	6				4	5	5	12	1	26.37	2
382	6				4	5	5	14	2	26.37	2
383	6				4	5	5	15	1	22.90	2
384	6				4	5	5	15	2	23.71	2
385	6				4	5	5	14	1	16.90	2
386	6				4	5	5	14	2	25.10	2
387	6				4	5	5	16	1	20.50	2
388	6				4	5	5	13	2	17.94	2
389	6				4	5	5	13	2	25.51	2
390	6				3	4	2	15	1	18.12	2
391	6				3	4	2	15	1	17.57	2
392	6				3	4	2	14	1	17.67	2
393	6				3	4	2	14	1	24.09	2
394	6				3	4	2	14	1	16.86	2
395	6				3	4	2	14	1	19.85	2
396	6				3	4	2	17	2	19.26	2
397	6				3	4	2	14	1	23.50	2
398	6				4	5	5	14	2	18.94	2
399	6				3	4	2	16	1	20.50	2
400	6				9	9	9	14	2	26.24	2
401	6				3	4	2	14	2	18.37	2
402	6				4	5	5	14	2	17.45	2
403	6				4	5	5	14	2	21.34	2
404	6				4	5	5	12	1	22.31	2
405	6				3	4	2	15	1	16.14	2
406	6				3	4	2	15	2	18.06	2
407	6				3	4	2	15	1	21.34	2
408	6				3	4	2	15	2	18.65	2
409	6				4	5	5	14	2	16.85	2
410	6				3	3	2	15	1	21.75	2
411	6				4	5	5	14	2	24.03	2
412	6				4	5	5	12	2	15.53	2
413	6				4	5	5	14	1	19.46	2
414	6				3	3	2	15	2	19.91	2
415	6				3	4	2	13	2	14.31	2
416	6				4	5	5	14	1	23.61	2
417	6				3	4	2	14	2	17.22	2
418	6				3	4	2	15	1	21.55	2
419	6				3	4	2	15	1	22.89	2
420	6				3	3	2	16	1	25.01	2
421	3	12	10	3	3	4	2	13	2	22.22	1
422	3	16	21	12	2	3	2	13	1	19.26	1
423	3	13	0	10	2	3	1	13	1	24.03	1
424	5	9	13	10	2	3	1	14	1	28.67	1
425	5	8	13	4	3	3	2	13	2	16.21	1
426	3	7	1	8	1	2	1	13	2	25.00	1
427	5	20	29	15	4	5	5	13	2	19.50	1
428	5	1	5	2	3	4	2	13	2	15.77	1
429	3	18	33	18	1	2	1	13	1	21.33	1
430	3	20	35	14	1	1	1	13	1	18.22	1
431	5	14	19	14	9	9	9	13	2	18.69	1
432	3	16	18	10	1	2	2	13	2	21.38	1
433	5	12	4	9	3	3	2	14	2		1
434	3	20	13	18	3	3	2	13	2	25.87	1
435	3	8	8	8	3	4	2	13	2	21.76	1
436	5	15	27	10	3	4	2	14	1	18.33	1
437	3	11	13	5	2	3	2	13	1	24.30	1
438	5	11	7	7	3	3	2	13	2	16.50	1
439	5	12	25	13	3	4	2	15	2	18.59	1
440	3	14	24	18	1	1	2	14	1		1
441	3	13	23	19	3	2	2	14	1	19.38	1
442	3	5	4	5	1	2	1	14	2	21.48	1
443	3	13	30	20	3	3	2	13	1	23.78	1
444	5	7	9	10	2	3	2	13	2	18.69	1
445	3	14	13	15	4	5	5	13	1	16.88	1
446	6	14	0	8	2	2	2	13	1	23.23	2
447	6	18	35	19	1	2	1	14	2	30.22	2
