# Supplementary material for: Undiagnosed prediabetes in Mexican adolescents under poverty in contexts affected by collective violence: A clinical comparison among health services users and hidden population
Source: Front Nutr. 2022 Nov 21;9:1007781. doi: 10.3389/fnut.2022.1007781 (PMC9720165; doi:10.3389/fnut.2022.1007781)
Supplement: Supplementary file 3 [file Data_Sheet_2.pdf]

Subject	Diagnosis	Sex	Age	Previous DX	AgeDX	Duration	BMI	GIH	HOMA	Cortisol	Carential	Context	Violence	Beck	RESI	AccessHS	Services	Socprog	Urban	Poberty	HealthS	Group
1	2	2	14	1	8	72	33.09	5.7	2.1	11.3				29		1	1	0	1	1	4	4
2	3	2	14	2	14	1		5.6	2.4	15.2				17	87			0	1	1	1	1
3	2	2	17	1	10	96	24.25	6.6	0.2	9.9				8		1	3	0	1	1	4	4
4	3	1	15	2	15	1	48.96	5.8	2.2	6.9				36	76	1	2	0	1	2	4	1
5	3	2	13	2	13	1	22.22	5.7	1.3	16.6	10	3	12	9	84	1	1	0	3	3	2	1
6	3	1	14	2	14	1		5.7	2.0	6.8									9	9	9	1
7	3	1	13	2	13	1	19.26	5.8	2.0	8.7	21	12	16	13	84	1	1	0	2	3	2	1
8	3	1	13	2	13	1	24.03	5.8	5.2	8.8	0	10	13	30	77	1	1	1	2	3	1	1
9	5	1	14	2			28.67	5.6	2.0	6.2	13	10	9	13	85	1	1	1	2	3	1	2
10	4	2	15	2	15	0		5.6	12.1	5.4									9	9	9	1
11	5	2	13	2	13	0	16.21	4.6	1.6	8.8	13	4	8	18	90	1	1	1	3	2	4	2
12	2	2	15	1	14	12	20.32	10.7	4.9	11.7				26		1	1	0	2	3	1	4
13	3	2	13	2	13	1	25.00	5.9	2.5	5.8	1	8	7	8	101	1	2	0	1	2	1	1
14	5	1	14	2					1.5	20.2									9	9	9	2
15	5	2	13	0			19.50	5.1	1.4	6.5	29	15	20	20	66	1	1	0	4	5	5	2
16	5	2	13	0			15.77	5.3	0.9	12.1	5	2	1	4	99	1	3	2	3	4	2	2
17	3	1	13	2	13	1	21.33	6.0	2.4	5.5	33	18	18	14	95	1	2	0	1	2	1	1
18	3	1	13	2	13	1	18.22	5.9	1.4	12.8	35	14	20	14	102	1	2	0	2	1	1	1
19	2	1	14	1	14	1	18.08	13.1								1	1	0	1	2	1	4
20	3	1	15	1	14	18	40.53	5.6	4.9	11.8				31		1	2	0	2	3	3	4
21	5	2	13	0			18.69	5.6	0.5	1.1	19	14	14	5	84	1	1	1	9	9	9	2
22	2	1	11	1	0	132	24.72	5.8	1.2	8.6				4		1	1		1	1	2	4
23	5	1	13	0				5.1	1.0	6.8									9	9	9	2
24	5	2	14	0			31.80	5.4	2.6	7.5						1	2		2	3	3	2
25	3	2	13	2	13	0	21.38	5.9	1.4	13.1	18	10	16	14	97	1	3	0	1	2	2	1
26	5	1	17	2	14	36	20.16	5.6	2.8	13.2				5	96	1	1	1	1	1	2	2
27	3	2	15	2	15	12	33.02	5.8	0.0					23	78	1	3	2	1	2	2	1
28	3	1	14	2	14	1		5.8	4.0	15.0										9	9	1
29	5	1	14	0				5.4	2.7	5.0				33	77				2	3	1	2
30	5	2	14	0				5.2	2.0	7.5	4	9	12	25	78	1	2	0	3	4	2	2
31	3	2	13	2	13	1	25.87	5.8	1.1	8.6	13	18	20	24	79	1	1	0	3	4	2	1
32	5	1	15	0			19.68	5.6	1.5	13.2				3	102				2	2	2	2
33	2	1	14	1	13	12	41.53	6.4	20.1	10.8				23		1	1		2	3	3	4
34	2	1	18	1	15	36	28.24	6.2	0.5	7.9				24		1	1	0	3	2	2	4
35	4	1	13	0	13	1								15	103				2	3	1	1
36	3	2	15	2	15	1		5.9						15	90	1	2	0	2	2	2	1
37	5	2	13	0			21.76	5.4	3.7	6.1	8	8	8	0	84	1	2	1	3	4	2	1
38	5	2	14	0				5.4								1	1	1	1	2	1	2
39	5	1	13	2	13	0		5.5	7.5	5.5										9	9	1
40	3	1	10	1	11	0	31.83	5.7	13.9	11.2	9	7	13	7	95	1	2	0	1	2	4	4
41	5	1	14	0			18.33	5.4	1.6	8.4	27	10	15	20	91	0	0	0	3	4	2	2
42	3	2	14	1	13	18	39.97	5.9						37	71	1	2	0	1	2	4	4
43	5	1	13	2	13	0		5.5	1.8	12.1									9	9	9	2
44	3	2	14	2	13	1		5.8	1.1	13.6				17	82	1	3	0	2	3	2	1
45	3	1	13	2	13	0	24.30	5.9	5.7	8.4	13	5	11	9	61	1	2	0	2	3	2	1
46	5	2	13	0			16.50	5.6	0.8	8.1	7	7	11	1	102	1	1	1	3	4	2	2
47	5	2	15	0			18.59	5.3	1.0	12.0	25	13	12	6	86	1	1	0	3	4	2	2
48	2	2	12	1	9	36	20.36	5.7	2.3	8.8				19		1	2	0	1	2	4	4
49	2	2	16	1	9	96	24.72	7.8	1.7	12.4	3	5	2	3	91	1	2	0	2	3	2	4
50	3	1	14	2	14	1		5.8	3.2	7.0	24	18	14	11	103	1	3	0	1	1	2	1
51	3	1	14	2	14	1	19.38	5.9	1.0	5.7	23	19	13		91	1	1	1	2	2	2	1
52	5	1	13	2	13	0		5.4	1.5	10.1				39	102			1	2	2	1	2
53	3	2	14	2	14	1	21.48	5.9	2.7	6.8	4	5	5	15	103	1	2	0	1	2	1	1
54	5	2	13	0			35.09	5.6								1		0	2	3	3	2
55	5	1	13	0				5.5	1.0	10.3				20	83	1	1	1	2	2	2	2
56	5	1	13	0			23.70	5.4	3.8	5.9	30	20	13	33		1	1	1	3	3	2	1
57	5	2	13	0			18.69	5.1	1.3	6.8	9	10	7	1	92	1	2	0	2	3	2	2
58	3	1	13	2	13	1	16.88	5.8	3.9	13.3	13	15	14	7	95	1	1	1	4	5	5	1
