# Supplementary figures and images for: Undiagnosed prediabetes in Mexican adolescents under poverty in contexts affected by collective violence: A clinical comparison among health services users and hidden population
Source: Front Nutr. 2022 Nov 21;9:1007781. doi: 10.3389/fnut.2022.1007781 (PMC9720165; doi:10.3389/fnut.2022.1007781)

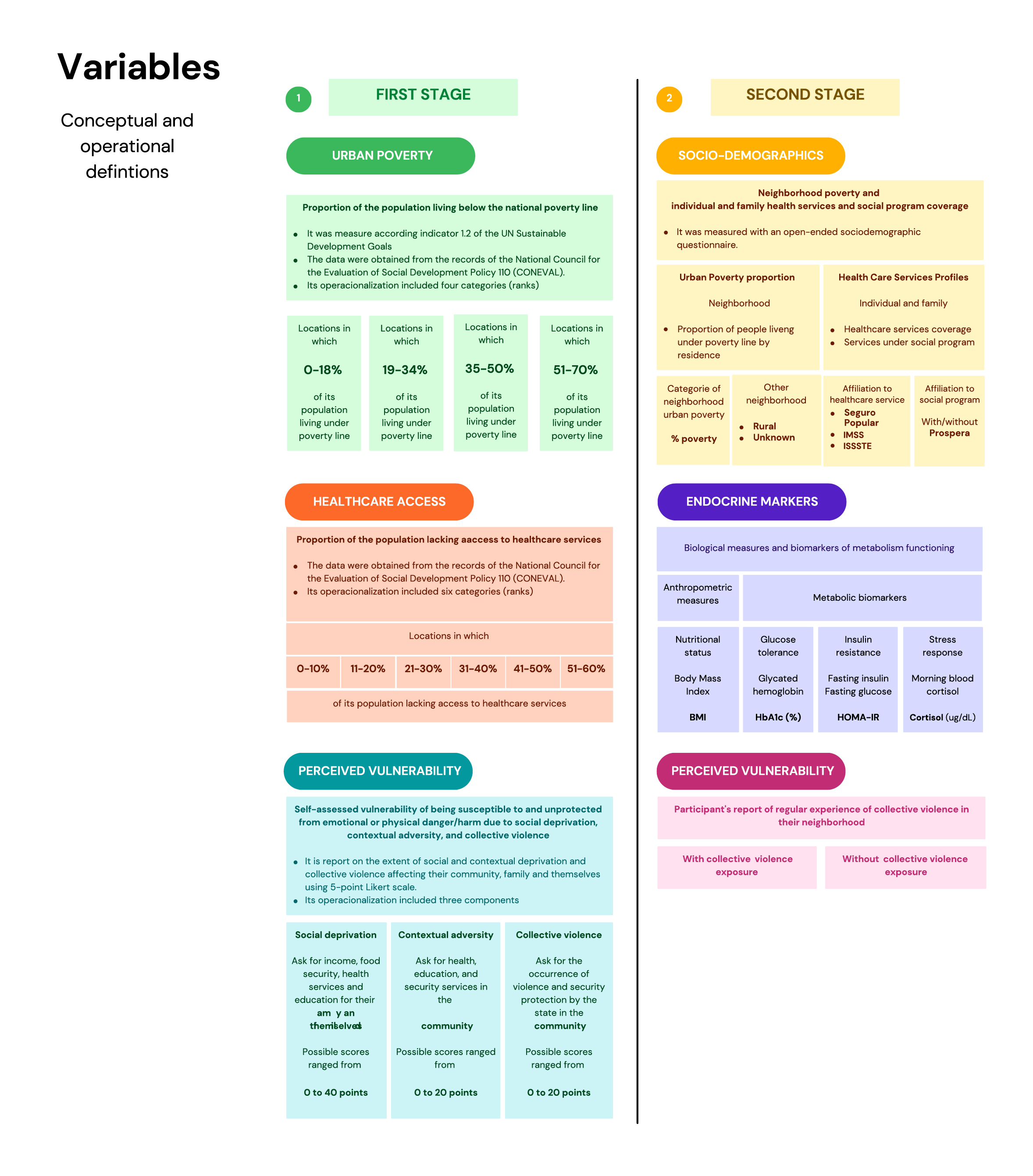

Supplement: Supplementary file 4 [file Image_1.JPEG]
